# No Clinically Relevant Memory Effects in Perinatal Hyperglycemia and Hypoglycemia: A 40-Year Follow-Up of a Small Cohort

**DOI:** 10.3389/fpubh.2022.858210

**Published:** 2022-07-01

**Authors:** Ilkka Järvinen, Jyrki Launes, Jari Lipsanen, Maarit Virta, Ritva Vanninen, Eliisa Lehto, Nella Schiavone, Annamari Tuulio-Henriksson, Laura Hokkanen

**Affiliations:** ^1^Faculty of Medicine, Department of Psychology and Logopedics, University of Helsinki, Helsinki, Finland; ^2^University of Eastern Finland, Institute of Clinical Medicine, Radiology, Kuopio, Finland; ^3^Department of Clinical Radiology, Diagnostic Imaging Center, Kuopio University Hospital, Kuopio, Finland

**Keywords:** gestational diabetes, perinatal hypoglycemia, diabetes complications, birth risks, cognitive functioning, memory, follow-up studies

## Abstract

Maternal diabetes mellitus in pregnancy is associated with impairments in memory functions of the offspring in childhood and adolescence but has not been studied in adulthood. The association of perinatal hypoglycemia with memory has not been studied in adulthood either. The combined sequelae of these two risk factors have not been directly compared. We studied general cognitive ability and memory functions in a prospective follow-up of a cohort born in 1971 to 1974. The sample included participants exposed to prenatal hyperglycemia (*n* = 24), perinatal hypoglycemia (*n* = 19), or both (*n* = 7). It also included controls with no early risks (*n* = 82). We assessed the participants' Intelligence quotient (IQ), working memory, and immediate and delayed recall of both verbal and visual material at the age of 40. We did not find significant differences in IQ or the memory tests between the groups. We did identify an interaction (*p* = 0.03) of the early risk with the type of digit span task: compared to the controls, the participants exposed to perinatal hypoglycemia had a larger difference between the forward digit span, a measure of attention, and the backward digit span, a measure of working memory processing (*p* = 0.022). The interaction remained significant when birth weight was controlled for (*p* = 0.026). Thus, in this small cohort, prenatal hyperglycemia, perinatal hypoglycemia, and their combination appeared relatively benign disorders. The association of these conditions with neurocognitive impairments in adulthood remains unconfirmed. The significance of the working memory difference needs to be verified with a larger sample.

## Introduction

### Prenatal Hyperglycemia

Maternal diabetes mellitus (DM) causing fetal and perinatal hyperglycemia may be associated with congenital malformations and neurological problems in the offspring (1). An association of maternal DM and the offspring's long-term cognitive impairment has been suggested in several studies (2–4). However, the results are contradictory, as intellectual quotient (IQ) scores higher than in controls have also been found in the offspring of diabetic mothers (ODM) (5). Existing follow-up studies extend to childhood or early adulthood and have included subjects who had also concomitant birth risks.

In a study of 3- and 4-year-old ODM, performance was impaired in both immediate and delayed explicit recall of events (6), although these findings were interpreted to be associated with iron deficiency. A comparison of 10-year-old ODM and controls found no group-level differences in memory (7).

In a group of forty 6- to 12-year-old OMD, the Working Memory Index of the Wechsler Intelligence Scale for Children (WISC) (8) was low as compared to WISC normative data and other indices of WISC (9). However, this study had no control group. In another study of over thirty 7- to 9-year-old ODM, the Working Memory Index was lower than in controls (10). In a group of over two hundred 13- to 19-year-old ODM, a composite score compiled from two of Reynolds Intellectual Assessment Scales was lower than in controls (11).

Maternal DM was associated with a slightly worse overall cognitive performance in a cohort of two hundred 18- to 20-year-old male ODM who were compared to 700 male controls. In an intelligence test performed by military conscripts (12), no group difference was found, but in a subgroup of 40 subjects, unsatisfactory maternal blood glucose indicated by high HbA1c values was negatively associated with their test performance. Likewise, in a subgroup of 60 ODM, maternal fasting glucose levels were negatively associated with intelligence test performance.

A study of over 720.000 men undergoing an army conscript suitability examination found an association of maternal DM and lower offspring IQ at the age of 18 years (13). The study also assessed cognitive function within sibships discordant for maternal DM. In this population, no difference was found. The authors conclude that rather than a perinatal mechanism, shared familial characteristics may explain the association between maternal DM and cognitive function.

Differences in cognitive performance between 18- to 27-year-old ODM and controls have been found in two studies, but these (2, 14) assessed general intelligence, and memory was not specifically examined.

We found no published data on whether the cognitive impairments remain after early adulthood. Animal models demonstrate abnormal hippocampal development (15, 16) and impaired learning of tasks (17). Based on such possibility of effect on the hippocampal formation, we regard it a plausible hypothesis that memory deficits could be observed in adulthood.

### Perinatal Hypoglycemia

Symptomless low blood glucose levels may occur in healthy newborns (18), but long-lasting perinatal hypoglycemia may cause permanent brain damage (19, 20). Concerns about the harmfulness of moderate hypoglycemia rose based on the findings of a multi-center study in 1988 (21), which found impaired mental and motor development at 18 months. Those findings have since been refuted, but no consensus yet exists either on the cut-off limit or duration of a harmfully low blood glucose, because confounding conditions such as low birth weight and gestational age modify the harmfulness of hypoglycemia (22). Furthermore, infants of diabetic mothers may have had hypoglycemia *in utero* and therefore the classification may be inaccurate.

In a study (22) of 35 rather severely affected children with hypoglycemia and concomitant acute neurological findings, 94% had mild to severe diffuse white matter abnormalities and 51% had cortical abnormalities in brain MRI during the first postnatal week. At age 18 to 24 months, the neurodevelopmental status was normal in 8, mildly abnormal in 15, and severely abnormal in 11 out of 34 subjects.

In another study of 404 children with transient postnatal hypoglycemia, no association with unfavorable neurodevelopmental outcome was found at the age of 2 years (23) or 9–10 years (24). Impaired executive and visual motor functions were seen in this cohort at the age of 4.5 years (25) but no longer at 9–10 years (24). The cohort included infants who were at risk of neonatal hypoglycemia but who were screened and treated if needed. Therefore, not finding differences between groups in this study may reflect the success in preventing complications by means of systematic monitoring and rapid intervention, rather than the effects of hypoglycemia *per se*. This study also enrolled infants who had concomitant conditions and risk factors, including hyperglycemia. Although the most evident confounding factors were included in the statistical analysis, it may not have been able to detect all differences in outcome.

A study of over 500 children (26) with transient mild (<1.95 mmol/L) postnatal hypoglycemia found a relatively strong association of hypoglycemia with lower school achievement at the age of 10. However, this study included newborns of any gestational age and excluded the 34 infants who had prolonged hypoglycemia.

In a 15-year follow-up study (27) of 47 children with recurrent but asymptomatic and relatively mild hypoglycemia (<2.5 mmol/ L), neither impaired cognitive nor motor development was found. However, also this study included subjects with a variety of confounding conditions.

In two studies (2, 14), perinatal hypoglycemia was found to be unassociated with a global cognitive score in ODM. In another study, the functioning of memory in ODM was significantly impaired after controlling for perinatal complications including hypoglycemia (9). No study, however, has specifically investigated the association of cognitive performance with combined hyperglycemia and hypoglycemia.

We aimed to study memory functions in adults who had had prenatal hyperglycemia, prolonged perinatal hypoglycemia, or a combination of them as the only perinatal risks of cognitive impairment.

## Methods

### Participants and Follow-Up

The subjects participated in a prospective follow-up study, which is described in detail elsewhere (28–31), of 1,196 newborns born between 1971 and 1974 with various predefined risk factors in a single maternity unit in Helsinki, Finland. They have been followed up at 5, 9, 16, 30, and 40 years of age. For the purposes of the present study, only subjects with prenatal hyperglycemia, perinatal hypoglycemia or both were included. Subjects that had coinciding perinatal risks, such as low birthweight, hyperbilirubinemia or low Apgar scores indicating asphyxia, were excluded. Prenatal hyperglycemia was diagnosed by at least two abnormally high glucose levels in morning urine samples during the regular pregnancy monitoring visits of the mother, an abnormal glucose tolerance test of the mother, or pre-existing diabetes of the mother. Hypoglycemia was diagnosed if the newborn had at least twice a serum glucose level below 1.67 mmol/L (full-term newborns) or below 1.21 mmol/L (infants <37 gestational weeks). Twenty newborns had hypoglycemia in addition to prenatal hyperglycemia. Hypoglycemia was diagnosed during the first day *postpartum* and treated in all cases, mainly by increasing the frequency of feeding.

The control subjects, also prospectively studied from childhood, were born in the same hospital during the study period, attended the same schools, and had no perinatal risk factors.

**Figure 1:**
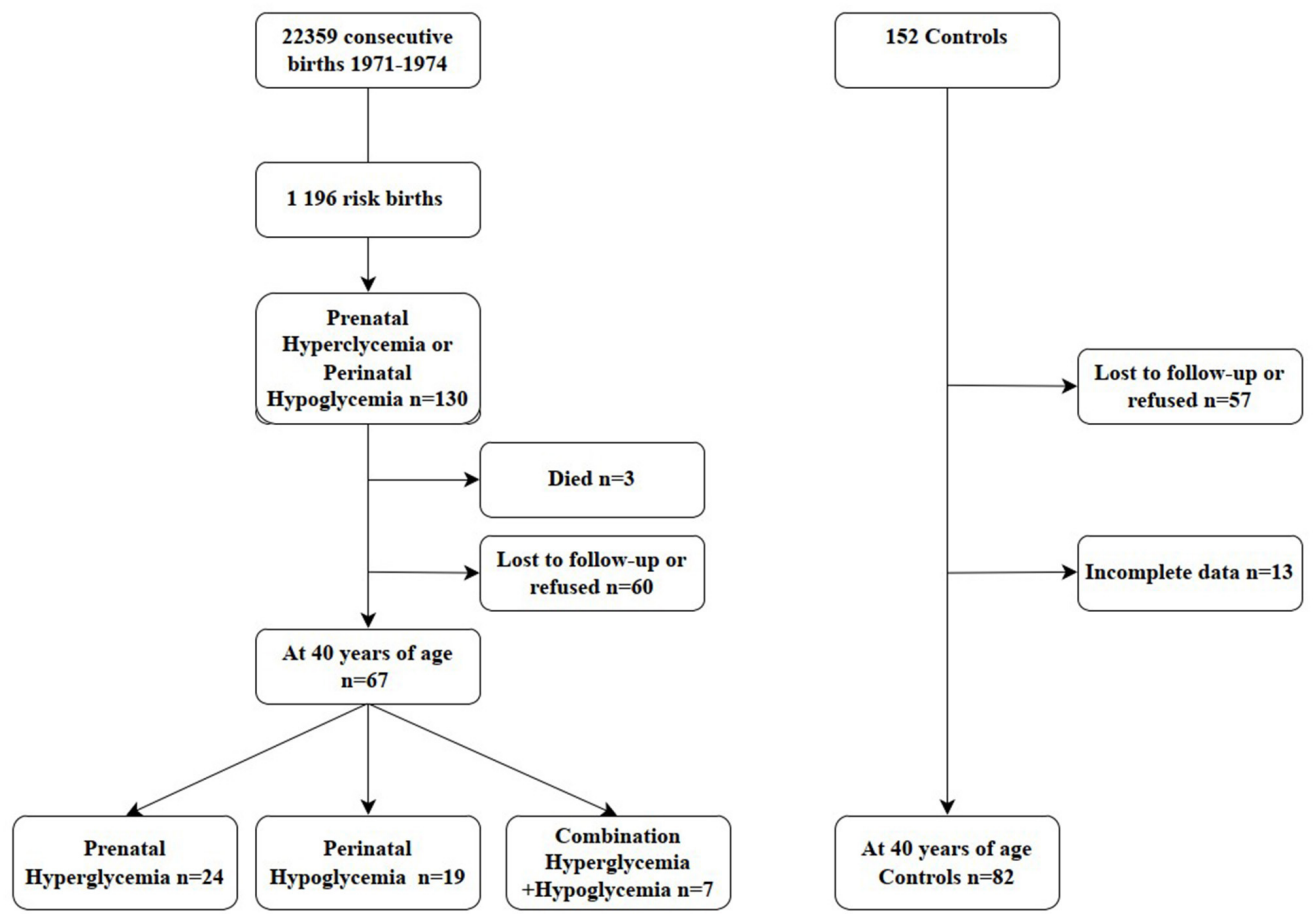
The flow of participants in the birth-risk cohort and controls.

The final groups analyzed comprised 132 subjects. They were classified into four groups: (1). subjects who had been prenatally exposed to high maternal blood glucose level (prenatal hyperglycemia *n* = 24), (2). subjects who had low perinatal blood glucose level (perinatal hypoglycemia *n* = 19), (3). subjects who had both prenatal hyperglycemia and perinatal hypoglycemia (combined risks *n* = 7), 4. controls (*n* = 82). A flowchart of the inclusion and exclusion of subjects is given in Figure 1.

Neurological development at 5 years of age was examined with the Neurodevelopmental Test (NDT) (32), which is a localized version of the test developed by Bax and Whitmore (33). It assesses a wide range of neurodevelopmental areas, including perception, motor abilities, and behavior.

The family's socio-economic status (SES) was estimated using the highest level of either parent's occupation at follow-ups at 0, 5, 9, or 16 years. The two lowest-level categories of the original four-level classification were combined due to the small number of subjects in the lowest category. At 9 years, information was collected from the parents regarding special education (targeting reading, spelling, mathematics, speech and language, or any combination of these) provided in collaboration with schools. Education level was defined as the highest completed education level reported by the subject at age 40.

### Attrition

Attrition was analyzed using logistic regression with separate models for five predictors: (1). group membership (risk or control) in the base cohort, (2). gestational age, (3). birth weight, (4). family socioeconomic status (SES, defined as the highest status at 0, 5, 9, or 16 years), and (5). Full-Scale IQ (FSIQ) at 9 years of age, measured by the Finnish version of WISC (34). To investigate potential differences in attrition patterns between risk and control groups, models 2 to 5 were refitted with group membership as an additional predictor. Regardless of risk status, subjects in the lowest SES category were less likely to participate than those in the highest SES category (OR = 0.35, 95% CI 0.19–0.66, *p* = 0.001). Subjects with a higher IQ were more likely to participate regardless of risk status (OR = 1.05 per one IQ point, 95% CI 1.02–1.07, *p* < 0.001). Birth weight only predicted participation when risk status was controlled for (OR = 1.10 per 100 g, 95% CI 1.02–1.19, *p* <0.021). Exposure to birth risks or gestational age did not predict participation.

### Assessment of General Cognitive Ability and Memory Functions

General cognitive ability at age 9 was assessed by IQ of the Finnish version of WISC (34) and at age 40 by the FSIQ of the Finnish version of the Wechsler Adult Intelligence Scale–Fourth Edition (WAIS-IV) (35). The FSIQ was calculated from seven subtests: Similarities, Vocabulary (every other item), Information, Block Design, Matrix Reasoning, Digit Span, and Coding. Memory functions were assessed with the Digit Span subtest of WAIS-IV and Logical Memory and Word List subtests of the Finnish version of the Wechsler Memory Scale–Third Edition (WMS–III) (36), and the Rey-Osterrieth Complex Figure Test (37). Neuropsychological tests were completed in one session with the investigators blinded to clinical data.

### Clinical and Neuroradiological Evaluation

Subjects were evaluated neurologically by a neurologist (JL). A total of 122 subjects had also a 1.5 T brain MRI scan, including a T1-weighted sequence, the T2-weighted fluid-attenuated inversion recovery (FLAIR), diffusion imaging, and T2-star weighted angiography (SWAN). Scans were read blindly without any knowledge of clinical data by an experienced specialist of neuroradiology (RV).

### Statistical Analyses

To avoid most violations of assumptions required by traditional parametric methods, we calculated means and bootstrapped confidence intervals with bias correction and acceleration (10,000 resamples) and *p* values with permutation tests (10,000 permutations). The memory tests were analyzed with ANOVA between-by-within models with the different sub-tasks of each test as levels of the within-subjects factor. For the Digit Span test, the different tasks (Digit Span Forward, Digit Span Backward, and Digit Span Sequencing) served as levels of the within-subjects factor. In the remaining tests, the different trials served as levels of the within-subjects factor: the immediate and delayed trial in the Logical Memory test; the four learning trials, the post-interference-list trial, and the delayed-recall trial of the Word List (thus excluding the interference-list trial); and the immediate and delayed reproduction trials of the Complex Figure Test. Pairwise comparisons were also calculated with permutation tests insensitive to departure from normality. *P* values of multiple comparisons were adjusted with Holm's sequential Bonferroni method. The analyses were conducted using the statistics software R (38).

## Results

No differences were found in the categorical background variables (sex, SES, special education received, education level) between the groups (Table 1), nor in IQ at age 9 or the age at the time of this study (Table 2). However, there was a difference in birth weight and NDT. In pairwise comparisons, the hypoglycemia group had a significantly lower birth weight than all other groups (hypoglycemia vs. hyperglycemia and hypoglycemia vs. controls, *p* < 0.001; hypoglycemia vs. combined risk, *p* = 0.001); no other pairwise comparisons of birth weight were significant. To account for a potential confounding effect of birth weight, it was used as a covariate in the analysis of the memory measures. In pairwise comparisons of the NDT, the hyperglycemia (*p* < 0.001) and combined risk (*p* = 0.005) groups had a higher impairment score than the control group; no other differences were significant.

**Table 1:** Categorical variables: sex, education, family socioeconomic status, and incidental findings in adulthood (count and percentage).

**Demographic characteristic**	**Total** **sample (*n* = 132)**	**Prenatal hyper-** **glycemia (*n* = 24)**	**Perinatal hypo-** **glycemia (*n* = 19)**	**Combined risk** **(*n* = 7)**	**Controls** **(*n* =82)**	***P*** **^e^**
Sex						0.595
Female	70 (53%)	12 (50%)	8 (42%)	5 (71%)	45 (55%)	
Male	62 (47%)	12 (50%)	11 (58%)	2 (29%)	37 (45%)	
Family SES^a^						0.264
High	54 (41%)	8 (33%)	8 (42%)	1 (14%)	37 (45%)	
Middle	55 (42%)	12 (50%)	5 (26%)	4 (57%)	34 (42%)	
Lower middle and low	23 (17%)	4 (17%)	6 (32%)	2 (29%)	11 (13%)	
School special education received at 9 years^b^						0.257
No	73 (64%)	11 (58 %)	7 (47%)	3 (50%)	52 (70%)	
Yes	41 (36%)	8 (42%)	8 (53%)	3 (50%)	22 (30%)	
Level of education completed^c^						0.070
Basic education (compulsory, 9 years)	7 (5%)	1 (4%)	4 (21%)	0 (0%)	2 (2%)	
Secondary education (typically 12 years)	64 (49%)	15 (65%)	7 (37%)	3 (50%)	39 (48%)	
Higher education	59 (45%)	7 (31%)	8 (42%)	3 (50%)	41 (50%)	
MRI incidental findings^d^	19 (15 %)	2 (9 %)	3 (18 %)	0	14 (18 %)	0.722

*SES, Socioeconomic status*.

a *The highest of the values reported when the participant was 0, 5, 9, or 16 years*.

b *Self-reported. The value was missing for two participants (1.10%)*.

c *Special education data available for 114 cases*.

d *MRI data available for 122 cases*.

e *Generalization of Fisher's exact test to r × c contingency tables*.

**Table 2:** Continuous background variables: birth weight, neurodevelopmental test at 5 years, cognitive performance at 9 years, and age at time of study.

**Measure**	**Total sample**		**Prenatal hyperglycaemia**		**Perinatal hypoglycaemia**		**Combined risk**		**Controls**		***p*** **^b^**
	***n***	***M*** **[95% CI]**	***n***	***M*** **[95% CI]**	***n***	***M*** **[95% CI]**	***n***	***M*** **[95% CI]**	***n***	***M*** **[95% CI]**	
Birth weight (g)	132	3,503 [3,410, 3,594]	24	3,769 [3,534, 3,995]	19	2,743 [2,523, 3,006]	7	3,790 [3,436, 3,968]	82	3,576 [3,502, 3,664]	<0.001
NDT^a^ at age 5	71	13.7 [11.8, 16.2]	21	17.6 [14.3, 23.2]	16	13.1 [9.7, 17.6]	6	22.7 [13.4, 34.3]	28	9.2 [7.5, 11.3]	0.001
IQ at age 9	95	120.9 [118.5, 123.4]	20	118.1 [112.3, 123.9]	15	119.6 [113.2, 125.3]	6	116.7 [102.5, 131.5]	54	122.9 [119.7, 126.0]	0.349
Age	132	41.4 [41.2, 41.6]	24	41.4 [40.9, 41.9]	19	41.1 [40.6, 41.6]	7	42.0 [41.0, 42.7]	82	41.5 [41.2, 41.7]	0.429

*The confidence intervals are bootstrapped with 10,000 iterations. NDT, Neurodevelopmental Test; IQ, Intelligence Quotient measured by Wechsler Intelligence Scale for Children*.

a *Error score*.

b *Permutation test with 10,000 permutations*.

### MRI

No major brain MRI abnormalities were found among the 122/132 subjects imaged, but there were several incidental findings, two of which required referral. Among the 22 hyperglycemic subjects one subject had a right frontal developmental venous malformation and another had megacisterna magna. Among the 17 hypoglycemic subjects, two had microbleeds (1 hemispheral, 1 cerebellar) and one had a developmental venous malformation. No abnormalities were found in the combined risk group (*n* = 5). In the control group there were 14 / 78 subjects with incidental findings (4 microbleeds, 4 developmental venous malformations, 2 arachnoidal cysts, a cavernoma, a colloid cyst, a small meningioma, a cyst of the choroidal fissure, and a hypophyseal macroadenoma). The frequency of incidental findings did not differ between the groups (Table 1).

### Memory Domains

There were no significant differences between the groups in FSIQ or any memory test (Table 3). However, the group-by-trial interaction was significant in the Digit Span test. This was further examined by pairwise comparisons of the interaction between the groups: the only significant comparison was that between the hypoglycemia and control groups (*p* = 0.022). These groups were compared for each of the three differences between the three trials: after correcting for multiple comparisons, the only significant comparison was in the difference between Digit Span Forward and Digit Span Backward (*p* = 0.002). The group-by-trial interaction in the Digit Span test remained significant after adjusting for the effect of birth weight (*p* = 0.026).

**Table 3:** Means and confidence intervals of the memory tests and results of the group comparison.

**Test**	**Total sample (*n* = 132)**	**Prenatal hyperglycemia** **(*n* = 24)**	**Perinatal** **hypoglycemia** **(*n* = 19)**	**Combined risk** **(*n* = 7)**	**Controls** **(*n* = 82)**	**Group comparison**	
Test	***M*** **[95% CI]**	***M*** **[95% CI]**	***M*** **[95% CI]**	***M*** **[95% CI]**	***M*** **[95% CI]**	*Effect*	*p^a^*
FSIQ	112.0 [109.0, 114.8]	110.0 [102.0, 116.9]	108.1 [96.3, 115.6]	110.7 [98.3, 121.5]	113.6 [110.1, 116.7]	Group	0.560
Digit Span						Group Task Group by task	0.691 <0.001 0.03
Forward	6.4 [6.2, 6.5]	6.5 [6.1, 7.0]	6.7 [6.3, 7.0]	5.7 [5.1, 6.1]	6.3 [6.0, 6.5]		
Backward	5.6 [5.3, 5.8]	5.8 [5.2, 6.2]	5.1 [4.5, 5.4]	5.1 [4.0, 5.9]	5.6 [5.3, 6.0]		
Sequencing	6.3 [6.1, 6.6]	6.5 [5.9, 7.1]	6.4 [5.7, 6.8]	6.4 [5.1, 7.1]	6.3 [6.0, 6.6]		
Logical Memory						Group Task Group by task	0.470 <0.001 0.811
Immediate	15.4 [14.7, 16.1]	16.1 [14.4, 17.4]	14.4 [11.9, 16.1]	14 [12.0, 17.9]	15.6 [14.7, 16.4]		
Delayed	13.5 [12.8, 14.2]	14.0 [12.4, 15.3]	12.7 [10.3, 14.3]	12.7 [10.1, 16.4]	13.6 [12.7, 14.5]		
Word List						Group Task Group by trial	0.862 <0.001 0.824
Trial 1	6.0 [5.7, 6.3]	5.8 [5.1, 6.3]	5.6 [5.1, 6.1]	5.7 [4.3, 6.7]	6.1 [5.8, 6.5]		
Trial 2	8.5 [8.2, 8.8]	8.5 [7.8, 9.1]	8.3 [7.1, 9.0]	8.7 [7.7, 9.3]	8.6 [8.1, 9.0]		
Trial 3	9.7 [9.4, 10.0]	9.8 [9.0, 10.2]	9.2 [8.1, 9.8]	9.6 [9.0, 10.1]	9.8 [9.4, 10.1]		
Trial 4	10.2 [9.9, 10.4]	10.3 [9.7, 10.7]	9.7 [8.8, 10.4]	10.6 [9.6, 11.3]	10.2 [9.8, 10.5]		
Trial 5	8.3 [7.9, 8.7]	8.8 [7.7, 9.4]	8.0 [6.7, 8.8]	9.3 [8.0, 10.0]	8.2 [7.7, 8.7]		
Delayed	8.0 [7.5, 8.4]	8.2 [7.3, 8.9]	7.8 [6.4, 8.9]	8.7 [7.4, 9.6]	7.9 [7.3, 8.4]		
Complex Figure Test						Group Task Group by task	0.776 <0.001 0.847
Copy	33.8 [33.3, 34.1]	33.7 [32.9, 34.3]	33.5 [32.4, 34.3]	33.7 [31.9, 35.0]	33.8 [33.2, 34.3]		
Immediate	23.5 [22.4, 24.6]	24.3 [21.1, 26.6]	21.9 [19.0, 24.1]	22.7 [19.7, 25.3]	23.7 [22.2, 25.0]		
Delayed	22.6 [21.5, 23.7]	23.0 [19.7, 25.4]	21.6 [18.4, 24.0]	21.7 [18.7, 23.8]	22.9 [21.3, 24.2]		

a *Permutation test with 10,000 permutations. FSIQ = Full-Scale IQ*.

## Discussion

We did not find significant differences in general intelligence or several memory tests in our small cohort of healthy adults with prenatal hyperglycemia or perinatal hypoglycemia as their single or combined risk. The only finding was a group-by-task interaction on the Digit Span subtests of WAIS-IV related to a larger difference between the Digit Span Forward and Digit Span Backward in the hypoglycemia group compared to the controls. This may indicate difficulty in working memory processing. There were no clinically significant brain MRI findings in any of the groups. Some minor incidental findings were observed in all groups, including controls. There was no difference in the education level of the groups reached by the age of 40.

### Memory Performance in Prenatal Hyperglycemia

We did not observe associations between prenatal hyperglycemia and memory functioning in adulthood. Two studies report working memory impairment in childhood (12, 13), and one study on adolescent children reports impairment in a composite memory index (11). An additional study on infants reports impaired memory, but this finding was based on event-related potential studies and the implications remain uncertain (39). However, not all studies have found an association between maternal diabetes and childhood memory (7). It is therefore unclear if no differences in adulthood indicate a problem that has been attenuated by adulthood, or a lack of a problem to begin with.

In comparing the results, several considerations are in order. Most importantly, although our study group is a part of a large cohort, it is undeniably small and has therefore many of the restrictions of a case series. However, we did have the advantage of a control group. There are also other differences between the samples and study designs. Our sample consisted of relatively mild cases because mothers at a significant delivery complication risk, i.e., most mothers with DM1, were not included. However, not all mothers in our sample received treatment for DM, and gestational DM was detected in a delayed manner during pregnancy in many cases. Thus, we lacked individuals resembling participants in a study that reported impaired working memory, in which all participants were born to mothers with poor adherence to treatment of diabetes (10). Our sample was demographically homogenous and recruited from a single hospital, whereas many studies do not describe the background characteristics in detail, e.g., another recent study (11) included deliveries in eight different centers but possible center effects were not taken into account. An attrition analysis of our population indicated that participants with a lower IQ were more likely to be lost to follow-up. Attrition was not analyzed in most of the previous studies (7, 9, 10), which causes concerns for generalizability.

Previous studies have found an effect of maternal DM on global cognitive function in young adulthood, between 18 and 27 years (2, 12, 40). We found no difference in IQ between ODM and subjects with no early risks at the age of 40 in our small cohort. As in the case of memory functions, the discrepancy may be caused by differences between study samples and selection. The previous studies used registry data of pre- and perinatal events. Two studies (12, 13) included male offspring only, and one (13) did not address the possible center or area effects, even though the participants were from the whole country of Sweden, and the time span is 20 years, which is a long time considering the rapid developments in diabetes care.

### Memory Performance in Perinatal Hypoglycemia

We found no unequivocal association between perinatal hypoglycemia and memory or global cognitive functioning in adulthood. We did observe an interaction in the subtype of the span task which was due to a difference between the group with perinatal hypoglycemia and the controls. In the hypoglycemia group, the difference between the forward and backward span was larger than in the control group. This may suggest a subtle impairment in verbal working memory associated with neonatal hypoglycemia: the backward-span task requires effortful processing in working memory (41), and this appears impaired compared to the efficiency of attention measured by the simple forward-span task. The clinical relevance of this finding is unclear and requires larger samples to be verified.

The lack of a more extensive association between perinatal hypoglycemia and memory functioning is in line with two (24, 27) of the previous studies conducted in childhood and adolescence. The most recent study included also measures of spatial working memory and visual paired associate learning at age 9–10 (24). Lower academic achievement at the age of 10 in children with transient perinatal hypoglycemia has been reported in one study (26), with a considerably higher cutoff value (2.5 mmol/L) than in our study (1.67 mmol/L). Stricter criteria for hypoglycemia were used in the 1970s than in many of the other studies (23, 26, 27, 42). It is also uncertain if memory test measurements and academic achievement can be directly compared. In the present study, with the small sample size, no difference was found in the educational level achieved by the age of 40 between the study groups. In sum, while we recognize that severe long-lasting hypoglycemia may have serious consequences, our results support the conclusion that treated perinatal hypoglycemia is not a significant risk for impairment of cognitive functions.

### Memory Performance in Combined Prenatal Hyperglycemia and Perinatal Hypoglycemia

We found no clear association of combined perinatal hyperglycemia and perinatal hypoglycemia with memory functioning in adulthood. Three studies of children of diabetic mothers (2, 9, 14) which analyzed perinatal hypoglycemia reported no association with cognitive impairment. In sum, results suggest that the combined effect of perinatal hypoglycemia and hyperglycemia would be no larger than that of prenatal hyperglycemia only.

### Strengths and Limitations

Our study assesses the influence of hyperglycemia (gestational DM) and hypoglycemia more specifically than other studies on this subject, as we did not include subjects who had other birth risks that could affect cognition, e.g., low birth weight, low Apgar score, and hyperbilirubinemia. Another major strength is the prospective longitudinal design. As the participants have been followed up from birth to adulthood, we were able to verify clinically and radiologically that the subjects did not have concomitant neurological or cognitive disorders. The prospective design also allowed for analysis of attrition quantifying the sampling bias. Although our sample was biased toward participants with a higher level of cognitive functioning, there is no indication that the magnitude of the difference would be dissimilar in the different groups.

Another strength of this study is the use of robust resampling methods, which are more powerful than non-parametric rank-based methods. These robust methods enable more accurate analyses than normality-based methods, which are sensitive to violations of assumptions in small and unequal samples. Violations lead especially to a risk of false positive results.

An undeniable limitation is the small size of the groups. In that respect the study can be considered a case series, although the initial cohort of the longitudinal follow-up was sizeable. The data collected in childhood regarding cognitive performance, family environment and special education provided was incomplete which hampers longitudinal multivariate analyses. The robust statistical methods that we employed should reduce some of the problems related to small samples, i.e., loss of power and the risk of false positive results. Nevertheless, these methods cannot completely remedy the problems of statistical inference when groups are small and unequal in numbers. The results need to be interpreted with caution.

## Summary and Conclusions

We found no indication that either maternal DM or perinatal hypoglycemia associate with clinically relevant global cognitive impairment, memory impairment, or brain MRI changes in adulthood. Subtle differences in working memory processing were found associated with perinatal hypoglycemia but they were not linked with cognitive impairment in this small cohort, and their significance needs to be verified with a larger sample.

## Data Availability Statement

The datasets presented in this article are not readily available because restrictions apply to the data. The ethics review board decision demands confidentiality. Pseudonymized data are available to a qualified investigator from the corresponding author. Requests to access the datasets should be directed to LH, laura.hokkanen@helsinki.fi.

## Ethics Statement

The studies involving human participants were reviewed and approved by the Ethical Review Board of the Helsinki and Uusimaa Hospital District. The patients/participants provided their written informed consent to participate in this study.

## Author Contributions

IJ collected data, carried out the analyses, drafted the initial manuscript, and reviewed and revised the manuscript. JLa, LH, and MV conceptualized and designed the study, coordinated and supervised data collection, collected data, and critically reviewed the manuscript for important intellectual content. JLi carried out the analyses, reviewed, and revised the manuscript. EL, AT-H, and NS collected data, reviewed, and revised the manuscript. RV analyzed, interpreted data, reviewed, and revised the manuscript. All authors approved the final manuscript as submitted and agree to be accountable for all aspects of the work.

## Funding

The Finnish Cultural Foundation, Päivikki and Sakari Sohlberg Foundation, and Finnish Brain Foundation (IJ); Juho Vainio Foundation (IJ and LH); Social Insurance Institution of Finland (Kela), the Diabetes Research Foundation, Jalmari and Rauha Ahokas Foundation, Yrjö Jahnsson Foundation, and Signe and Ane Gyllenberg Foundation (LH). Helsinki University Library provided funding for the open access publication.

## Conflict of Interest

The authors declare that the research was conducted in the absence of any commercial or financial relationships that could be construed as a potential conflict of interest.

## Publisher's Note

All claims expressed in this article are solely those of the authors and do not necessarily represent those of their affiliated organizations, or those of the publisher, the editors and the reviewers. Any product that may be evaluated in this article, or claim that may be made by its manufacturer, is not guaranteed or endorsed by the publisher.
